# Seasonal Pattern of *Mycobacterium ulcerans*, the Causative Agent of Buruli Ulcer, in the Environment in Ghana

**DOI:** 10.1007/s00248-017-0946-6

**Published:** 2017-02-25

**Authors:** Samuel Yaw Aboagye, Kobina Assan Ampah, Amanda Ross, Prince Asare, Isaac Darko Otchere, Janet Fyfe, Dorothy Yeboah-Manu

**Affiliations:** 1 Bacteriology Department, Noguchi Memorial Institute for Medical Research, University of Ghana, P.O. Box LG 581, Legon, Accra, Ghana; 2 Institute of Environmental and Sanitation Studies, University of Ghana, Accra, Ghana; 3 Swiss Tropical and Public Health Institute, Basel, Switzerland; 4 University of Basel, Basel, Switzerland; 5 Victorian Infectious Diseases Reference Laboratory, Melbourne, VIC Australia

**Keywords:** *Mycobacteriumulcerans*, Buruli ulcer, Ghana

## Abstract

This study aimed to contribute to the understanding of *Mycobacterium ulcerans* (MU) ecology by analysing both clinical and environmental samples collected from ten communities along two major river basins (Offin and Densu) associated with Buruli ulcer (BU) at different seasons. We collected clinical samples from presumptive BU cases and environmental samples from ten communities. Following DNA extraction, clinical samples were confirmed by IS*2404* PCR and environmental samples were confirmed by targeting MU-specific genes, IS*2404*, IS*2606* and the ketoreductase (KR) using real-time PCR. Environmental samples were first analysed for IS*2404*; after which, IS*2404*-positive samples were multiplexed for the IS*2606* and KR gene. Our findings indicate an overall decline in BU incidence along both river basins, although incidence at Densu outweighs that of Offin. Overall, 1600 environmental samples were screened along Densu (434, 27 %) and Offin (1166, 73 %) and MU was detected in 139 (9 %) of the combined samples. The positivity of MU along the Densu River basin was 89/434 (20.5 %), whilst that of the Offin River basin was 50/1166 (4.3 %). The DNA was detected mainly in snails (5/6, 83 %), moss (8/40, 20 %), soil (55/586, 9 %) and vegetation (55/675, 8 %). The proportion of MU positive samples recorded was higher during the months with higher rainfall levels (126/1175, 11 %) than during the dry season months (13/425, 3 %). This study indicates for the first time that there is a seasonal pattern in the presence of MU in the environment, which may be related to recent rainfall or water in the soil.

**Electronic supplementary material:**

The online version of this article (doi:10.1007/s00248-017-0946-6) contains supplementary material, which is available to authorized users.

## Background

Buruli ulcer (BU), caused by *Mycobacterium ulcerans* (MU), is the third most important mycobacterial disease of public health importance globally after tuberculosis and leprosy [1]. The disease has been reported in 32 countries worldwide mostly in the tropical regions with the greatest burden experienced in West African countries along the Gulf of Guinea [2]. Buruli ulcer, which affects the skin and its underlying soft tissues, begins usually as a painless papule or nodule under the skin at the site of trauma, but in some individuals, more severe diffuse forms occur such as a plaque and/or oedema. Failure to treat these early forms results in gradual erosion of the skin leaving a well-demarcated ulcer with wide undermined edges resulting from the cytopathic action of the plasmid-encoded macrolide toxin, mycolactone [3–5].

The epidemiology of BU in endemic countries is not fully understood. It has a focal distribution of cases where endemic and non-endemic communities are separated by few kilometres [6]. Nevertheless, various studies have linked high BU incidence to slow-flowing or stagnant waters and disturbed environment [7, 8]. Rapid changes in landscape [9] such as deforestation, flooding, construction of dams and artificial lakes for irrigation, mining activities and extending swamps for growing rice and fish breeding have been associated with the emergence of the disease in some communities [8, 10–13].

One of the factors limiting the prevention and control of BU is the lack of understanding of the ecology and mode of transmission of the causative agent. Many features of MU ecology, including distribution within the environment, niche adaptation and host range(s), are still not fully known [9, 14–16]. Theories that have been proposed to explain the mechanism of MU transmission include (1) inhalation of aerosolized MU from contaminated water [5], (2) acquisition of MU through an insect or vector bite [5] and (3) contamination of an existing wound or site of trauma by the environment such as soil, vegetation and water among others [17–20]; however, none of these theories have been confirmed.

A major factor that has limited the understanding of MU ecology is the inability to culture viable organisms from the environment [10, 20, 21]. However, the completion of the MU genome sequence provided specific targets for DNA-based detection methods, such as the insertion sequence IS*2404*, IS*2606* and the plasmid encoding mycolactone, ketoreductase-B domain (KR) [22]. Such methods have been used to elucidate tree-dwelling possums and mosquitoes as possible reservoirs [23] and potential vectors [24], respectively, in South-Eastern Australia. In a previous study, certain water bugs were cited as possible vectors in hosting MU in its salivary glands [25]; however, until now, no potential reservoirs have been identified in Africa, which harbours most of BU disease burden.

Seasonal changes are cyclic and represent a major source of external variation influencing human and other natural systems [26–30] and affect diseases such as malaria [31] and diarrhoea [32]. It is not clearly known whether the incidence of BU is seasonal. Understanding the local seasonal drivers of MU in the environment which could influence incidence of BU disease may be of importance in improving the control strategies in Ghana. In this study, we surveyed the presence of MU in different environmental sources using DNA-based assays and looked at seasonality and also possible risk factors within the environment and retrospectively characterized the occurrence of BU cases for each community based on active surveillance data.

## Methods

### Study Site

**Fig. 1:**
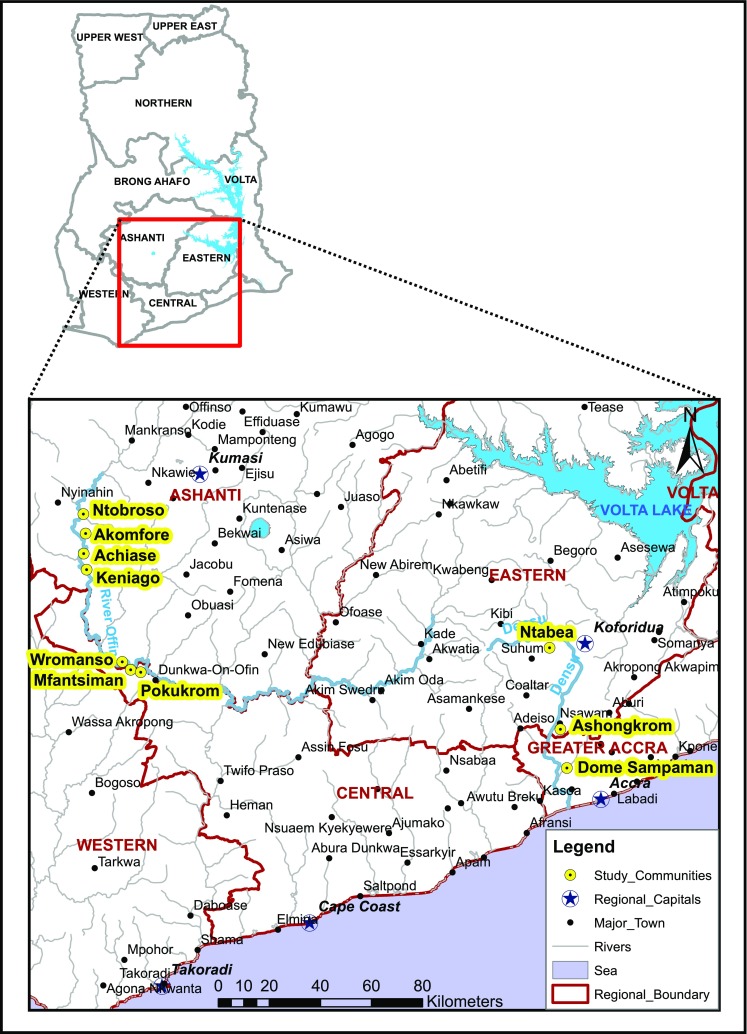
Map of Ghana showing study communities along the Densu and Offin River basins

The study was conducted in ten communities associated with BU along two major river basins (Densu and Offin) of Ghana (Fig. 1). These river bodies were selected for the study because extensive disease and sero-epidemiological studies have shown high exposure of community member to the *M. ulcerans* 18 KDa heat shock protein 65 [6, 33, 34], and unlike other communities in Ghana which depend on passive case report, these sites are active in reporting BU cases to the national BU control programme (NBUCP) [35].

Following the Ampah et al. [36] method, ten communities were selected by simple randomization from a total of 199 using a randomization tool embedded within the ArcGIS 10.0 software. Along the Densu River basin, the three randomly selected communities were Ntabea in the Akim East which lies upstream of the river, Ashongkrom in the Akwapim South at midstream both in the Eastern region and Domesampaman in the Ga-West Municipality of the Greater Accra region. In Offin, the seven randomly selected communities for the study were Ntobroso in the Atwima district which lies upstream of the river, Akomfore in the Amansie West district and Achiase, Wromanso and Keniago all in the Atwima district which lie midstream of the river and downstream of the river are Mfantsiman and Pokukrom in the Upper Denkyira district.

Sampling was conducted within the two major climatic seasons in Ghana, the rainy and dry season. The months of May to July are classified as the major rainy season and August to October as the minor rainy season with the remaining months constituting the dry season. The communities along the Densu River basin were predominantly hamlets with smaller coverage area, whilst those at the Offin River basin were large communities with extensive coverage area.

### Buruli Ulcer Active Case Search

We conducted active case search to monitor the emergence of BU cases using community outreach programme and monthly household visits by community volunteers. Community outreach education was conducted once every 3 months in all the selected communities. During the outreach programme, we educated community members on the transmission, early case detection and treatment of BU by showing BU documentaries and interacted with community members through questions and answers. The following morning, the inhabitants were screened and those with clinically suspected BU lesions were sampled for laboratory confirmation. We also employed a monthly household visit-based surveillance as an additional tool to the surveillance by the community outreach programme. We trained and equipped one community-based surveillance volunteer (CBSV) from each of the communities with android phones (HTC wildfire S) pre-loaded with a BU surveillance questionnaire. The questionnaire used for the study was designed as previously described [36]. Starting from August 2013 to December 2014, we mandated the CBSVs to visit all households monthly and record any presumptive case using the mobile application and a notebook. Presumptive cases were then sampled by a local health staff, and the samples were sent to the Noguchi Memorial Institute for Medical Research (NMIMR) for laboratory confirmation. We estimated the prevalence of BU within each of the communities using passive data obtained from the local health facilities and data from the national active case surveillance.

### Environmental Sample Collection

Two different sampling methods (convenient and random) were employed along the two river basins. Convenience sampling was conducted to collect environmental samples from the three communities (Ntabea, Ashongkrom and Domesampaman) along the Densu River basin in 2011, 2013 and 2014. We purposely used convenient sampling technique to capture specific zones within the environment where there is likelihood of human interactions. During the convenient sampling, we walked through the communities and collected environmental samples from sites of frequent human activities such as water sources including hand dug wells, ponds, streams and boreholes which are regularly utilized, communal bathing areas, school compounds, agricultural farms, market grounds and community centres. Samples were collected from 239 different locations, and at each sample location, we collected soil sample and any other sample within 1 m reach. Any other samples that were more than 1 m away from the location point were excluded from the collection. At each sampling location, a distance of about 10 m was allowed between sampling points or about 5 m where space was limited and GPS coordinates were taken at each collection point. Solid samples such as animal faeces (sheep, lizard and chicken), terrestrial insects (using insect net), snails, soil and water were collected aseptically into 50-ml Falcon tubes and vegetation parts were collected and pressed into 50-ml Falcon tubes and then released into sealable plastic bags from which biofilms were prepared. All samples were clearly labelled immediately, kept in a cool pack at 4 °C after collection and transported to the laboratory and kept frozen until analysis.

Along the Offin River basin, sampling was conducted in 2013 and 2014. Random sampling from a grid of locations was used for the collection of environmental samples from seven communities (Achiase, Akomfore, Keniago, Mfantsiman, Ntobroso Pokukrom and Wromanso). All the communities were mapped and divided into grids, and 487 sampling points were randomly selected using a randomization tool embedded within the ArcGIS 10.0. Samples were collected from each of the 487 points generated and treated in the same way as those from the Densu River basin and transported to the laboratory. We collected rainfall data from the Ghana Meteorological Agency, Accra after monthly rainfall level data from the meteorological substations within the study sites have been reported.

### Sample Processing

Snail and faecal samples were diced with sterile disposable surgical blades and homogenized using sterile porcelain and pestle and suspended in phosphate-buffered saline (PBS). Soil samples were shaken vigorously in sterile distilled water and centrifuged at 600 rpm for 5 min to sediment soil particles. Biofilms were prepared from vegetation parts using a modified version of the method described by Gryseels et al. [37]. Samples were emptied into sterile plastic resealable bags, and 50 ml of PBS was added to each bag. The contents of the bags were vigorously agitated to dislodge the biofilms into solution. The suspensions were poured into sterile 50-ml Falcon tubes and centrifuged at 4000 rpm for 30 min, to sediment all suspended bacteria. The supernatant was decanted and the resulting pellet was suspended in 10 ml of PBS for the analysis. Water samples were vortexed to mix homogeneously and centrifuged at 4000 rpm for 30 min to sediment all suspended bacteria. The supernatant was decanted and the resulting pellet was suspended in PBS.

### Screening of Samples by Real-Time PCR

Genomic DNA was extracted directly from 1600 environmental samples using the FastDNA SPIN kit for soil with the FastPrep-24^TM^ instrument (MP Biomedicals) according to the manufacturer’s instructions. Negative controls were included at each point of DNA extraction. Detection of MU DNA from the environment has been based solely on IS*2404* PCR due to the large copy numbers present in the MU genome [38]. However, there are other organisms that also harbour this IS*2404* sequence making it non-specific to MU [39]. In this study, three independent gene targets, IS*2404*, IS*2606* and KR, within the MU genome were screened. The extracted DNA was first screened for the insertion sequence IS*2404* by real-time PCR using Rotor Gene Q (Qiagen). Primers and TaqMan MGB probes from Applied Biosystems that were selected from regions of the sequences for IS*2404*, IS*2606* and KR present on the plasmid pMUM001 were used [22]. Probes IS*2404*TP and KRTP were labelled with the fluorescent dye 6-carboxyfluorescein (FAM) at the 5′ end and a nonfluorescent quencher at the 3′ end. Probe IS*2606*TP was labelled with the fluorescent dye VIC at the 5′ end and a nonfluorescent quencher at the 3′ end [22]. The IS*2404* real-time PCR mixtures contained 1 μl of template DNA, 0.9 μM concentrations of each primer, a 0.25 μM concentration of the probe, SensiFast (500 nM) mix (Bioline) and TaqMan exogenous internal positive control (IPC) reagents (Applied Biosystems) in a total volume of 20 μl. IS*2606* and KR assays were performed as a multiplex assay (without IPC) for all IS*2404*-positive DNA with CT value below 35. At each PCR run, two each of negative and positive controls were added. Amplification and detection were performed using the Gene Q sequence detection system (Qiagen) according to the following programme: 1 cycle of 50 °C for 2 min, 1 cycle of 95 °C for 15 min and 40 cycles of 95 °C for 15 s and 60 °C for 1 min. DNA extracts were tested in at least duplicate, and negative controls were included in each assay. All DNA samples that were positive for IS*2404*, IS*2606* and KR were classified as MU confirmed. We also determined the IS*2404*/IS2606 copy number ratio which differentiates *M. ulcerans* from the other mycolactone=producing mycobacterium following Fyfe et al. [22].

### Laboratory Confirmation of BU Cases

We confirmed presumptive BU lesions by collecting two swab specimens from the undermined edges of ulcerative lesions and one fine needle aspirate (FNA) collected into 500 μl PBS as previously described [40] for pre-ulcerative lesions. Samples were transported to NMIMR at 4 °C and confirmed by a positive IS*2404* PCR laboratory test as previously described [41].

### Statistical Analysis

The data collected were entered into a Microsoft Excel 2010 spreadsheet and analysed using R statistical software [42]. We used logistic regression and accounted for the cluster sampling by including random effects for location and community. We estimated the proportion positive with 95 % confidence intervals, overall and for different categories of the explanatory variables. In analyses stratified by site or adjusting for calendar month, the numbers were too small to allow the random effects model to converge and so we did not adjust for clustering.

## Results

### Demographic Characteristics and Identified BU Cases

An overall population of 10,851 inhabitants from communities along Densu (1217, 11.2 %) comprising 45.6 % (*n* = 555) females and 54.4 % (*n* = 662) males and Offin (9634, 88.8 %) comprising 49.6 % (*n* = 4785) females and 50.4 % (*n* = 4849) males were studied.

Within the study communities, we detected 84 presumptive BU cases; 56 (66.7 %) from Densu and 28 (33.3 %) from Offin River basins, respectively. Thirty-two (38.1 %) were laboratory confirmed by IS*2404* PCR, of which 24 (75 %) were from Densu and 8 (25 %) from the Offin River basin. At Densu, 15 (62.5 %) of the confirmed cases were from passive reports. The confirmed cases were detected in Ashongkrom (16, 66.7 %) and Domesampaman (8, 33.3 %) with a prevalence of 14.3 and 3.6 %, respectively, whilst no case was detected in Ntabea. At Offin, seven (87.5 %) of the confirmed cases were actively reported. The eight cases were detected in three communities: Achiase (4, 62.5 %), Ntobroso (3, 37.5 %) and Akomfore (1, 12.5 %). The recorded prevalence was 3.8 % for Achiase, 1.6 % for Ntobroso and 3.0 % for Akomfore (Table 1).

**Table 1:** Demographic characteristics and identified BU cases in studied communities

Variables	Community									
	Densu			Offin						
	A	B	C	D	E	F	G	H	I	J
Population (*n*)	378	512	327	1900	1016	3350	303	1949	900	216
No. of households	52	64	48	295	157	413	85	251	121	41
Sex: females, *n* (%)	184 (48.6)	228 (44.5)	143 (43.7)	939 (49.4)	499 (49.1)	1719 (51.3)	141 (46.5)	972 (49.9)	409 (45.4)	106 (49.1)
Presumptive BU cases	29	27	0	6	3	3	3	7	4	2
Lab confirmed	16	8	0	4	1	0	0	3	0	0
BU prevalence (%)	14.3	3.6	0	3.1	3	1.2	8.9	1.6	1.3	2.3

Prevalence rate is given as the total prevalence comprising both active and healed lesions

*A* Ashongkrom, *B* Domesampaman, *C* Ntabea, *D* Achiase, *E* Akomfore, *F* Keniago, *G* Mfanstiman, *H* Ntobroso, *I* Pokukrom, *J* Wromanso

Of the laboratory confirmed cases, 19 (59.4 %) were males and 13 (40.6 %) were females, aged between 3 and 70 years, with a mean age of 26. Lesions presented by cases were in the early stages; 25 (78.2 %) were detected with pre-ulcerative lesion and 7 (21.9 %) presented with category II lesions.

### Detection and Identification of *M. ulcerans* by Real-Time PCR

A total of 1600 environmental samples from ten communities associated with Buruli ulcer along both the Densu (434, 27 %) and the Offin (1166, 73 %) river basins were collected, categorized and screened for MU DNA using three independent molecular markers. We sampled from 239 locations in three communities along the Densu and 487 locations in seven communities in the Offin River basins. The median number of samples per location was 1 for the communities along the Densu River basin and 2 for those along Offin River basin. Overall, 139 (9 %) samples were positive for MU DNA (Table 2).

**Table 2:** *M. ulcerans* DNA positivity among samples analysed from communities along Densu and Offin River basins

Samples	Densu		Offin		Total MU confirmed	
	No. of samples	MU confirmed (%)	No. of samples	MU confirmed (%)	No. of samples	MU confirmed (%)
Detritus	2	1 (50.0)	21	0 (0.0)	23	1 (4.3)
Faeces	44	4 (9.1)	36	0 (0.0)	80	4 (5.0)
Fungi	8	1 (12.5)	3	0 (0.0)	11	1 (9.1)
Insect	8	1 (12.5)	28	0 (0.0)	36	1 (2.7)
Moss	35	7 (19.4)	4	1 (25.0)	40	8 (20.0)
Water	67	7 (10.4)	76	2 (2.6)	143	9 (6.2)
Soil	143	30 (20.9)	443	24 (5.2)	586	54 (9.0)
Vegetation biofilm	121	33 (27.5)	555	23 (3.9)	675	56 (8.1)
Snails	6	5 (83.3)	–	–	6	5 (83.3)
Total	434	89 (20.5)	1166	50 (4.2)	1600	139 (8.7)

We found MU DNA to be broadly distributed in all the communities along both river basins.

Two different sampling techniques were used in the study for both the Densu and Offin River basin. *M. ulcerans* positivity was significantly higher with the convenience sampling method conducted along the Densu River basin (89, 21 %) than the random sampling method at Offin (50, 4 %) river body (*p* < 0.001) (Table 2).

Along the Densu River basin, sample positivity for *M. ulcerans* DNA was high at Ntabea (21/37, 57 %), followed by Ashongkrom (61/293, 22 %) and the least at Domesampaman (7/104, 7 %) as shown in S1. Among the seven communities along the Offin River basin, Wromanso (16/142, 11 %) had the highest MU positivity and the least MU positivity was recorded for Akomfore (2/139, 1 %), Keniago (2/177, 1 %) and Pokukrom (2/88, 2 %), respectively (S1).

From samples collected along the Densu River basin, we found MU DNA positives among all nine sample types sampled with a positivity ranging from 83 % among snails to 9 % in animal faecal samples (Table 2). However, in the Offin River basin, detections were observed among only moss, soil, vegetation and water (Table 2). We detected MU in at least one sample each of vegetation and soil at every sampling period in both river basins. Among the samples confirmed to contain MU DNA, we found the highest proportion from agricultural farms (45 %), followed by water sources within the communities (36 %) and the least near household (19 %).

Of the 139 samples confirmed to contain MU DNA, the IS*2404*/IS*2606* copy number ratio, which differentiates *M. ulcerans* from the other mycolactone producing mycobacterium, was found in most of the samples to be around 2.4, the expected ratio for MU (S2). This IS*2404*/IS*2606* copy number ratios were found in 73/89 (88 %) and 37/50 (74 %) positive samples along the Densu and Offin River basins, respectively.

### Rainfall Pattern and MU Positivity

To better understand the seasonal drivers for the distribution of MU in the environment, we compared monthly MU positivity with monthly rainfall levels using data obtained from the meteorological substations within the study communities after approval from the Ghana Meteorological Agency, Accra.

We detected MU DNA in at least one sample in all the sampling periods throughout the study. The proportion of MU positive samples recorded was higher during the months with higher rainfall levels (126/1175, 11 %) than during the dry season months (13/425, 3 %; *p* < 0.001).

**Fig. 2:**
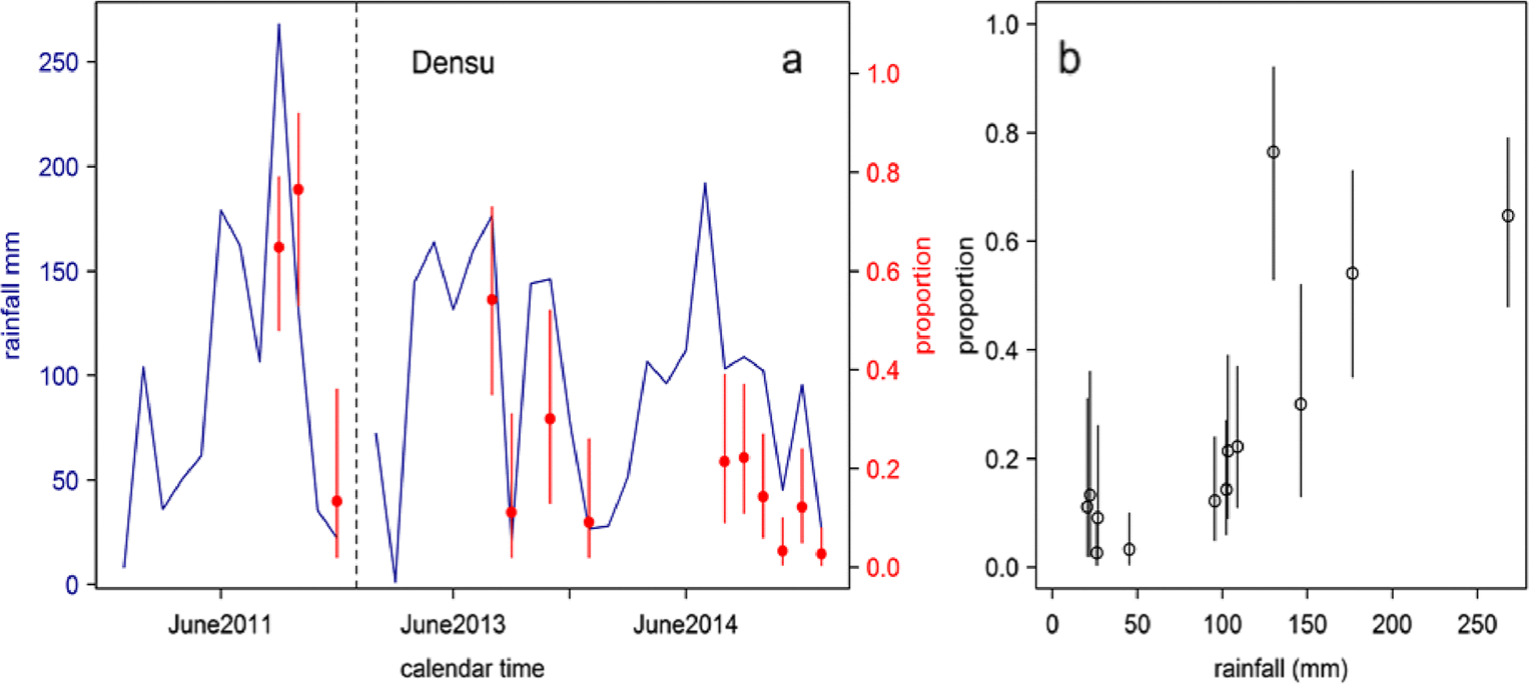
**a**, **b** Rainfall levels and *M. ulcerans* distribution along the Densu River basin

Along the Densu River basin in 2011, we recorded high MU positivity for the minor and major rainy seasons, September 23/34 (65 %) and October (13/17, 76 %), respectively, while MU positivity declined during the dry season, December (2/15, 13 %), as indicated (Table 3 and Fig. 2) (*R* = 0.94). In both 2013 and 2014, we observed a drastic decline in MU positivity in both the major and minor rainy seasons as well as the dry season (Table 3) (*p* = 0.0002, 95 % CI = 1.7–6.4).

**Table 3:** Rainfall levels and monthly MU DNA positivity along the Densu River basin

Period of sampling	Rainfall levels (mm)	Densu		
		No. of samples	MU positive	MU positivity rate (%)
September 2011	268.1	34	22	65
October 2011	130.1	17	13	77
December 2011	22.2	15	2	13
July 2013	176.6	24	13	54
August 2013	20.6	18	2	11
October 2013	146.2	20	6	30
December 2013	26.7	22	2	9
July 2014	103.3	28	6	21
August 2014	108.9	36	8	22
September 2014	102.4	42	6	14
October 2014	45.3	61	2	3
November 2014	95.5	41	5	12
December 2014	26.2	76	2	3
Total		434	89	20.5

**Fig. 3:**
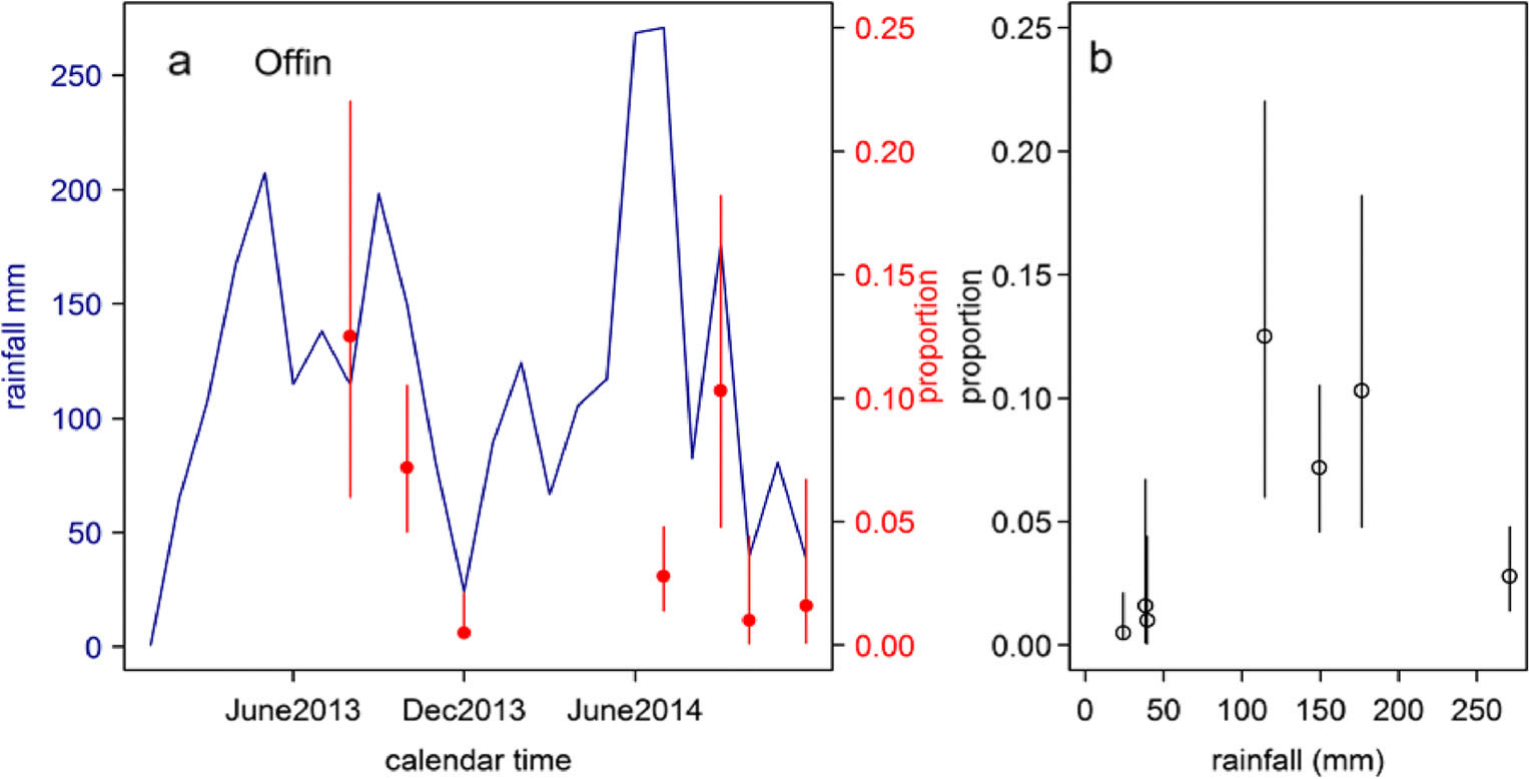
**a**, **b** Rainfall levels and *M. ulcerans* distribution in along the Offin River basin

Along the Offin River basin, our findings suggested that rainfall over a low threshold was associated with increased MU positivity (Table 4 and Fig. 3), but the linear trend observed in the Densu was not apparent. In both 2013 and 2014, MU positivity for the minor and major rainy seasons was also low as observed along the Densu River basin.

**Table 4:** Rainfall levels and monthly MU DNA positivity along Offin River basin

Period of sampling	Rainfall levels (mm)	Offin		
		No. of samples	MU positive	MU positivity rate (%)
August 2013	114.5	64	8	13
October 2013	149.4	292	21	7
December 2013	24.1	208	1	0.5
July 2014	270.9	292	10	2
September 2014	176.4	78	8	10
October 2014	39.5	98	1	1
December 2014	38.3	63	1	2
Total		1166	50	4.1

## Discussion

In this study, we looked for (1) the presence of MU DNA in the environment to contribute to understanding the pathogen ecology, (2) the associations between MU in the environment and other variables including BU endemicity and rainfall patterns and (3) the occurrence of BU cases within some selected communities. We found for the first time that there is a seasonal pattern in the presence of MU DNA in the environment, possibly related to rainfall and also more human BU cases are most likely to be detected after the raining seasons.

**Fig. 4:**
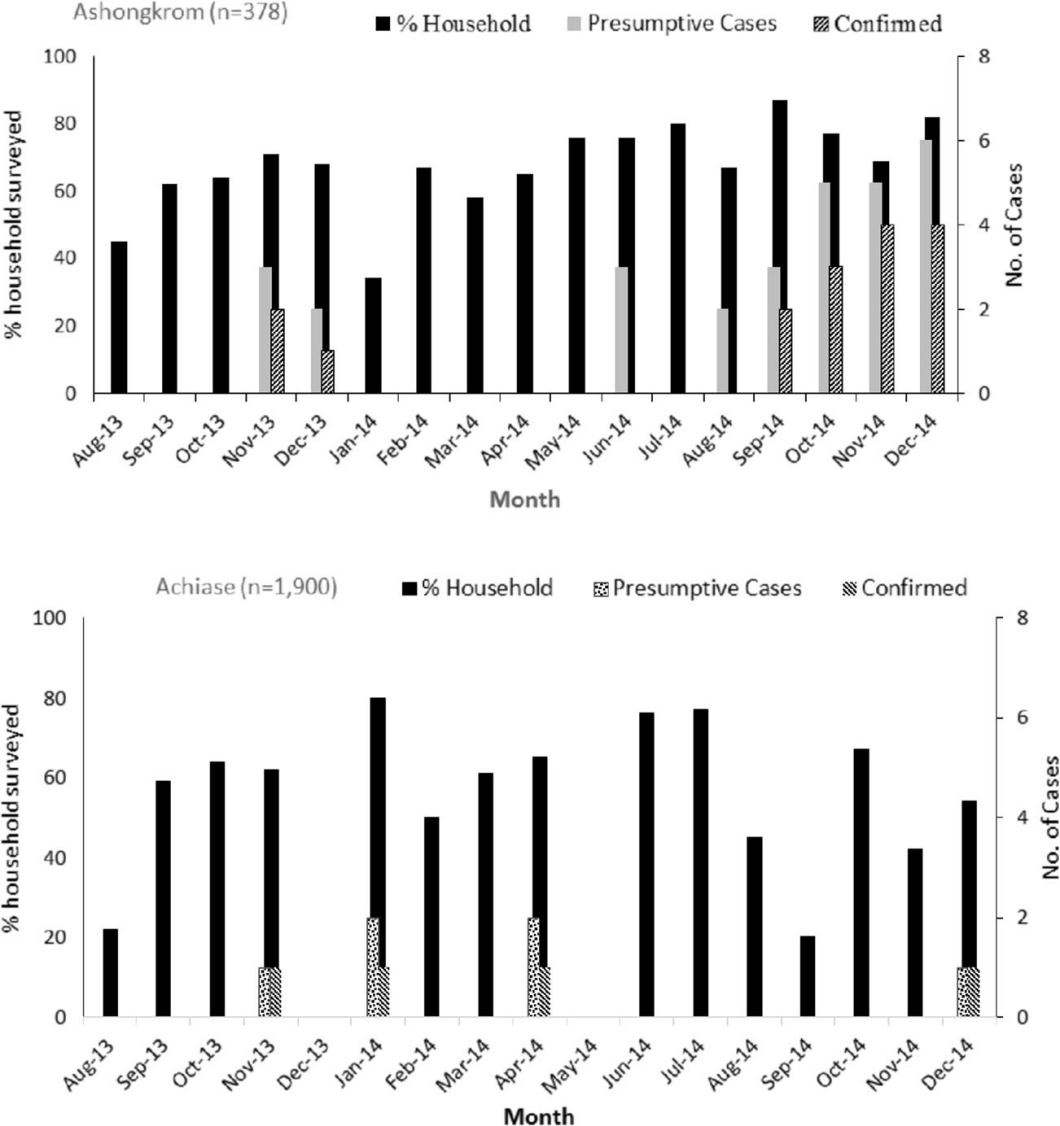
Monthly BU case surveillance for Ashongkrom and Achiase

A number of infectious diseases including vector, air and waterborne diseases are seasonal [26–29]. At present, there are no clear indications of association between BU and seasonality due to the long incubation period of the disease which is estimated between 2 and 4 months [43]. Nevertheless, few reports from some endemic countries indicate that there may be differences in occurrence of BU between wet and dry seasons in the tropics. In Cameroun and Papua New Guinea, high rates of BU occur during the dry season [44–46], while in both Ghana and Côte d’Ivoire, the peak incidence of the BU disease has also been reported to be at the end of the rainy season [47, 48]. Our finding therefore agrees with these reports as we detected more human BU cases during the monthly active case search surveillance conducted in Ashongkrom which peaked after the rainy months (Fig. 4). We are of the view that MU cells that might have been dormant/or buried in the environment particularly in the soil are exposed during the rainfall season due to the erosion and other environmental disturbances that occur during the rainfalls. The exposure rates that occur during these months probably account for the high numbers of BU cases at the end of the rainy season (in the BU treatment facility at the Amasaman Hospital, Accra, Ghana) or during the dry months due to increased agricultural activities, and this also supports the suggested incubation time [43–45, 47]. This same mechanism may account for the observation of high MU positivity rate from soil and vegetation biofilm samples that were detected at each sampling period (S2). Even though rainfall seems to influence the distribution of MU in the environment, our findings suggest that rainfall within a certain threshold was associated with MU positivity. This may mean that water in the ground is important probably for suspension but not so much water to wash away the bacteria.

*M. ulcerans* has been described as an environmental pathogen; this was confirmed in this study by finding MU DNA in all the communities along both river basins. The copy number ratios for the insertion sequences, IS*2404*/IS*2606*, for most of the positive samples were about 2.4. According to Fyfe et al., the average Δ*C*_T_ (IS*2606*–IS*2404*) for *M. ulcerans* is 2.37; while for the other mycolactone-producing mycobacteria, it is 7.60 [22]. We found 79 % of the analysed samples in the expected range for *M. ulcerans*, an indication that *M. ulcerans* was being detected (S2). However, the environmental abundance of MU seems to outweigh the observed disease occurrence. This could be due to the presence of non-human pathogenic strains of MU from the environment probably resulting from mixed populations of bacteria each carrying PCR targets but not necessarily MU itself. The broad distribution of MU in the environmental setting also clearly contrasts the focal distribution of human BU cases. Ntabea for instance has reported no BU case even though community members are exposed to the 18-kDa shsp-specific antibodies of MU [6]. The monthly active case surveillance confirmed this as we detected no human BU case during the study period; however, environmental MU presence was high. This may imply that community member has high inborn or genetic protection that has a reduced attack rate with BU. Furthermore, our finding suggests other pathogens and host variables may be important for the occurrence of the disease. Moreover, various studies by our group have shown that not all exposed individuals develop overt disease [6, 49]. The pathogen variables that will be interesting to explore will be the genomic difference that could lead to differences in virulence between isolates obtained from the study sites. Currently, methods for the in vitro isolation of MU from the environment have recently been published [50]. This will pave the way for comparative studies between environment and clinical samples.

Studies have postulated that MU may be preferentially adapting to specific ecological niches such as plant biofilms due to its inability to produce light inducible carotenoids that serve as a shield against incident sunlight [49]. In our study, we found high proportions of MU present in snail, moss, vegetation and soil along the Densu River basin whilst MU was more restricted to vegetation and soil at the Offin River basin sites. Aquatic snails have been reported to transiently harbour MU without offering favourable conditions for its growth and survival [51]. In contrast, the edible land African giant snail (*Achatina fulica*) from which we detected MU DNA can harbour metabolically active bacterial communities in its gut [52] during feeding on plants and soil [53, 54]. Considering the high MU positivity rate among the snail samples, a larger collection of snails from both river basins to further explore the presence of MU in the environment will be essential. Our finding therefore is consistent with earlier studies by Stinear et al. [49].

This study focused on the presence of MU in the environment rather than whether it was the source of human infection. However, case–control studies conducted in BU-burdened communities have identified wearing short and lower-body clothing while farming [55] as risk factors for BU and covering limbs during farming [56] as protective for BU. The proportions of vegetation biofilm and soil samples (S3) with confirmed MU DNA from this study may be relevant particularly for agricultural farmers in the tropics who often engage in activities without protective clothing due to high temperatures.

Also in most BU-burdened communities, children may swim in stagnant waters that collect during rainfall that might contain MU which may expose susceptible hosts to MU. The findings from our study confirm the presence of MU in the environment as the sero-epidemiological studies conducted along the river basins indicated that sera of individuals above age 5 contained significant amounts of 18 kDa shsp-specific antibodies of MU. This indicates exposure to MU from the environment as children below 5 years showed no immune response [6, 33, 34].

This study is limited by the use of two different sampling techniques for the two study sites which may have influenced the high positivity observed along the Densu site. However, we observed a similarity in higher positivity in both vegetation and soil samples irrespective of sampling technique, which again underpins the MU adaptation preferences for these particular sample types. The study is limited also by sampling during only certain months of the year. It would be desirable for sampling to cover a whole 12 months.

In conclusion, the study provides information on the presence of MU in the environment and for the first time indicates the influence of rainfall on its presence in the environment. In addition, we found clinical BU cases peaking after the raining seasons. We therefore recommend that further work on specific environmental sources may lead to human infection and potential protective measures are needed.

*BU* Buruli ulcer, *MU Mycobacterium ulcerans*, *PCR* polymerase chain reaction, *IPC* internal positive control, *PBS* phosphate-buffered saline, *NBUCP* National BU Control Programme, *CBSV* community-based surveillance volunteer

## Electronic supplementary material

ESM 1 (DOCX 14 kb)

ESM 2 (XLSX 20 kb)

ESM 3 (XLSX 13 kb)

ESM 4 (XLSX 18 kb)
