# Supplementary material for: Seasonal Pattern of Mycobacterium ulcerans, the Causative Agent of Buruli Ulcer, in the Environment in Ghana
Source: Microb Ecol. 2017 Feb 25;74(2):350–61. doi: 10.1007/s00248-017-0946-6 (PMC5496970; doi:10.1007/s00248-017-0946-6)
Supplement: Supplementary file 1 — (DOCX 14 kb) [file 248_2017_946_MOESM1_ESM.docx]

**S1.** *M. ulcerans* DNA positivity among study communities

| **Site** | **No. of Samples** | **MU Positive** | **MU Positivity Rate (%)** |
| --- | --- | --- | --- |
| ^‡^Ntabea | 37 | 21 | 57 |
| ^‡^Ashongkrom | 293 | 61 | 22 |
| ^‡^Domesampaman | 104 | 7 | 7 |
| *Achiase | 184 | 11 | 6 |
| *Akomfore | 139 | 2 | 1 |
| *Keniago | 177 | 2 | 1 |
| *Mfantsiman | 198 | 13 | 2 |
| *Ntobroso | 238 | 4 | 2 |
| *Pokukrom | 88 | 2 | 2 |
| *Wromanso | 142 | 16 | 11 |
| **Total** | **1600** | **139** | **8.7** |

^‡^Communities along the Densu river basin. *Communities along Offin river basin
